# Identifying subgroups with differential response to CBASP versus Escitalopram during the first eight weeks of treatment in outpatients with persistent depressive disorder

**DOI:** 10.1007/s00406-023-01672-0

**Published:** 2023-08-22

**Authors:** Ilinca Serbanescu, Elisabeth Schramm, Henrik Walter, Knut Schnell, Ingo Zobel, Sarah Drost, Thomas Fangmeier, Claus Normann, Dieter Schoepf

**Affiliations:** 1 Institute of Psychology, Heidelberg University, Hauptstrasse 47-51, 69117 Heidelberg, Germany; 2 Department of Psychiatry and Psychotherapy, Medical Center, Faculty of Medicine, University of Freiburg, Hauptstrasse 5, 79104 Freiburg, Germany; 3 Department of Psychiatry and Psychotherapy, Charité-University Medicine Berlin, Charitéplatz 1, 10117 Berlin, Germany; 4 Department of Psychiatry and Psychotherapy, University Medical Center Göttingen, Rosdorfer Weg 70, 37081 Göttingen, Germany; 5 Psychology School at the Fresenius University of Applied Sciences Berlin, Jägerstrasse 32, 10117 Berlin, Germany; 6 Department of Psychiatry and Psychotherapy, CBASP Center of Competence, University Medical Center Bonn, Sigmund-Freud-Strasse 25, 53127 Bonn, Germany

**Keywords:** Persistent depressive disorder, Personalized medicine, Escitalopram, CBASP, Moderator, Randomized clinical trial

## Abstract

There exists little empirical evidence helping clinicians to select the most effective treatment for individual patients with persistent depressive disorder (PDD). This study identifies and characterizes subgroups of patients with PDD who are likely to benefit more from an acute treatment with psychotherapy than from pharmacotherapy and vice versa. Non-medicated outpatients with PDD were randomized to eight weeks of acute treatment with the Cognitive Behavioral Analysis System of Psychotherapy (CBASP; n = 29) or escitalopram plus clinical management (ESC/CM; n = 31). We combined several baseline variables to one composite moderator and identified two subgroups of patients: for 56.0%, ESC/CM was associated with a greater reduction in depression severity than CBASP, for the remaining 44.0%, it was the other way around. Patients likely to benefit more from ESC/CM were more often female, had higher rates of moderate-to-severe childhood trauma, more adverse life events and more previous suicide attempts. Patients likely to benefit more from CBASP were older, had more often an early illness onset and more previous treatments with antidepressants. Symptomatic response, remission, and reductions in symptom severity occurred more often in those patients treated with their likely more effective treatment condition. The findings suggest that the baseline phenotype of patients with PDD moderates their benefit from acute treatment with CBASP relative to ESC/CM. Once confirmed in an independent sample, these results could serve to guide the choice between primarily psychotherapeutic or pharmacological treatments for outpatients with PDD.

## Introduction

Roughly 20–30% of patients with major depression develop a chronic course lasting two years or longer [1, 2]. In the fifth edition of the Diagnostic and Statistical Manual (DSM-5), this condition was first introduced as a distinct clinical category labelled as Persistent Depressive Disorder (PDD) [3]. Psychotherapy and pharmacotherapy, delivered as monotherapies or in combination, represent, in addition to brain stimulation, two main pillars of treatment for PDD, with the Cognitive Behavioral Analysis System of Psychotherapy (CBASP) [4] being the only psychotherapy model specifically developed to target PDD. CBASP has proven to be overall effective and has been thus recommended as first line psychotherapeutic treatment for PDD [5]. Nevertheless, various findings have shown that CBASP may not be the most effective treatment for all patients with PDD [6–9]. Similarly, the effect of pharmacotherapy appears to be limited for certain patients with PDD. For instance, a review by Kocsis [10] showed that the average rate of complete remission for patients with dysthymia and double depression was below 50% in several 6- to 12-week short-term studies.

Furthermore, pharmacotherapy was shown to be generally more effective (*d* = −0.31; 95% CI: − 0.53 to − 0.09) than psychotherapy in an earlier meta-analysis by Cuijpers and colleagues from 2010 [11]. However, this former result was exclusively due to patients with dysthymia included in the analysed studies, leaving open the question of which type of treatment works better for patients with other subtypes of PDD. In a more recent meta-regression by Furukawa and colleagues from 2018 [8], psychotherapy and pharmacotherapy showed similar results when delivered as monotherapies. However, this result was only valid for patients with characteristics near the population averages (e.g., low or moderate baseline depression and anxiety), with both monotherapies displaying different effects depending on the severity of baseline depression and anxiety, previous history of pharmacotherapy, age at baseline, and PDD subtypes. These results suggest that for some subgroups of patients, either CBASP or pharmacotherapy alone is a more effective treatment option and highlight the need for further investigations.

To date, there is little empirical evidence guiding clinicians to select the most effective treatment option for an individual patient with PDD [12, 13], and it is likely that the lack of personally selected and tailored treatment strategies is one of the main contributors to the overall low treatment success of patients with PDD [14]. In clinical practice, treatments for PDD are commonly selected in an unsystematic matter, often based on subjective clinical experience, treatment-preference of patients or trial-and-error approaches [12, 15, 16]. Over the last two decades, only a modest number of studies have aimed to gain a better understanding of which subgroups of patients with PDD are most likely to benefit from a particular psychotherapeutic or pharmacotherapeutic treatment. With regard to the choice between psychotherapy and pharmacotherapy, studies have analysed the impact of baseline severity of depression and anxiety as well as patients age [8], self-reported traumatic childhood experiences [17, 18], dysfunctional attitudes [19] and patients treatment preference [7, 20]. The results of the meta-regression by Furukawa and colleagues from 2018 [8] showed that for patients with elevated initial depression and anxiety scores, combination treatment of psychotherapy and pharmacotherapy was generally more effective than pharmacotherapy alone, which in turn was more effective than treatment with CBASP alone. In contrast, patients with moderate baseline depression scores and mild anxiety scores benefited equally well from combination treatment and treatment with CBASP alone, but less from treatment with pharmacotherapy alone. In addition, this study reported that monotherapy with antidepressants was more likely to be tolerated by younger patients with PDD, who had a lower dropout rate when treated with antidepressants compared to CBASP. Regarding the moderating effect of childhood traumatic experiences, different results were reported across studies: In a secondary analysis by Nemeroff and colleagues from 2003 [18], monotherapy with CBASP was shown to be superior to monotherapy with nefazodone in patients who reported childhood trauma. However, these findings could not be replicated in a later study by Bausch and colleagues from 2017 [17], who concluded that CBASP and escitalopram—a modern antidepressant delivered in combination with clinical management—were equally effective in treating patients with PDD and childhood trauma, with CBASP possibly having a longer treatment latency in these patients. Furthermore, conflicting results were also reported for the role of patients' treatment preference: While Kocsis and colleagues [7] reported in 2017 that patients preferring CBASP had better treatment outcomes when receiving CBASP than when receiving nefazodone, and vice versa, Steidtmann and colleagues [20] could not replicate this association in their study published in 2012. Finally, another study by Shankman and colleagues from 2013 [19] showed that higher baseline scores of dysfunctional attitudes were associated with a better response to pharmacotherapy compared to psychotherapy.

Despite yielding first interesting although not always replicable results, this previous evidence base comes along with several limitations: First, the individually examined baseline variables of these studies do not reflect the full individuality of a patient, who will have many other variables which are potentially critical to its treatment response that are not taken into account when focusing on a single variable. Second, individual baseline variables often have little effect size as moderators, which limits their practical relevance with regard to treatment selection in clinical practice [21]. Third, the composition of other unconsidered baseline variables in a clinical sample may influence the results of a stratifying predictor or moderator analysis, which may partly explain why previous secondary analyses have repeatedly produced contradictory results. Fourth, the existing evidence base may result in conflicting treatment recommendations for clinical practice. For example, to a patient reporting childhood trauma and a preference for antidepressant medication, one would recommend antidepressants over CBASP based on the findings by Kocsis and colleagues [7] and at the same time CBASP over antidepressants based on the findings by Nemeroff and colleagues [18]. Taken together, these factors complicate the evidence-based treatment selection for clinicians, and necessitate newer statistical approaches that capture the integral individuality of patients and use it to predict outcomes under different psychotherapeutic and pharmacological treatments.

The overarching aim of the present study was to address the question of which outpatients with PDD are, based on their multivariable baseline profile, more likely to benefit from psychotherapy with CBASP than from pharmacotherapy with escitalopram during the first eight weeks of treatment, and vice versa, thereby adding new findings to the existing body of evidence. In contrast to the studies summarized before, rather than examining single moderating baseline variables, we sought to exploratory identify subgroups of PDD patients with different treatment benefits using a modern composite moderator method together with machine learning that enable to simultaneously consider the treatment effect moderating role of multiple baseline variables. Our analyses were based on the data of a bi-centric randomized controlled trial (RCT) by Schramm et al. [22], who compared the effectiveness of CBASP to escitalopram, a well-tolerated standard selective serotonin reuptake inhibitor, combined with clinical management (ESC/CM) over 28 weeks in a sample of outpatients with PDD. The general findings showed that the clinician-rated depression scores decreased significantly after both eight and 28 weeks, however with no significant differences between the two treatment groups. Furthermore, in the original RCT, in case of non-improvement (defined as < 20.0% reduction in depression severity) after the 8-week acute treatment phase, the other treatment condition was augmented for the following 20 weeks of the extended treatment phase. Non-improvers to the initial treatment caught up with the initial improvers in terms of depression severity by the end of the extended treatment phase after being augmented with the respective other condition [22]. In conclusion, CBASP and ESC/CM appeared to be equally effective treatment options for chronically depressed outpatients in both the acute and extended treatment phase, whereas for patients who did not respond to their first treatment in the acute phase, augmentation with the other condition during the extended phase appeared to be effective in reducing depression severity.

The secondary analysis of this RCT presented in this paper was conducted to revisit this conclusion by examining whether, despite the reported general equivalence of the two treatments, there were in fact ‘hidden’ subgroups of patients who were likely to benefit more from CBASP than from ESC/CM and vice versa during the acute 8-week treatment phase. In addition, we investigated whether the initial lack of response in those patients augmented with the other treatment condition at week eight was because they did not receive their likely more effective treatment during the first eight weeks, and whether the observed improvement at week 28 was due to the augmentation with the treatment condition from which they would have likely benefitted more from the beginning of the treatment. By considering multiple baseline variables, our analysis methodologically extends an earlier, previously cited secondary analysis of this RCT by Bausch et al. [17] from 2017, who found that ESC/CM outperformed CBASP in patients with childhood trauma after the 8-week acute treatment phase and that both therapies were equally effective in patients with childhood trauma after the extended treatment phase. By addressing the research question of *‘what works for whom’* with regard to the choice between CBASP and pharmacotherapy with escitalopram for PDD, we pursued the overarching aim of adding more sustainable evidence that can help clinicians in choosing between psychotherapeutic and pharmacological treatments, thereby addressing the urgent need to advance personalized medicine for PDD [13].

## Materials and methods

The original study by Schramm et al. [22], on which this secondary analysis is based, was an evaluator-blind, parallel-design, 2-armed RCT conducted between 2008 and 2013 at two university medical centers in Germany. The study was approved by the institutional review boards and ethics committees at each site. Written informed consent was obtained from all participants before study enrollment. Study registration was performed at the University Register of Clinical Studies (No. 2007-006914-41) and at www.clinicaltrials.gov (No. NCT00837564). For details on participant inclusion and exclusion criteria, please refer to the main publication of the original trial [22].

### Study interventions

CBASP: The CBASP is a highly structured interpersonal learning approach that integrates behavioral, cognitive and, most importantly, interpersonal treatment strategies with personal disciplined involvement [9, 17]. Based on the assumption that early interpersonal trauma has led to dysfunctional mechanisms of derailed affective and motivational regulation and a reduction in perceived functioning, the overarching goal of CBASP is to help the patient to recognize the consequences of one’s own behavior for others, to reduce social fear in the interpersonal hot spot area, and to develop social problem-solving skills and empathy [23]. In the original RCT, in the initial acute treatment phase of eight weeks, two weekly CBASP sessions were conducted during the first four weeks and one weekly CBASP session during the last four weeks to ensure a minimum of 12 CBASP sessions. Study therapists were experienced and trained professionals, and all sessions were videotaped and viewed regularly by the supervisors.

ESC/CM: The second treatment condition in this RCT was escitalopram as a well-tolerated standard selective serotonin reuptake inhibitor with an excellent benefit/side-effect ratio [24]. A minimum of a 2-week washout period of the previous antidepressant medication was required for study participation, if indicated. The initial dose of escitalopram was 10 mg/day for the first week, which was increased to 20 mg/day in the following weeks. For patients experiencing dose-related side effects, their dose could be reduced to a minimum of 10 mg/day later. Clinical Management is a psychoeducative, supportive and empathic intervention including symptom management, monitoring of the medication and its possible side effects, providing hope, encouragement, and simple advice. The guideline based visits were conducted by senior psychiatrists or advanced psychiatric residents, taking place weekly during the acute phase, and were limited to 20 min [22].

### Study sample and analyzed baseline variables

Sixty patients including one non-starter who was excluded from the analyses were randomly assigned to receive treatment with CBASP (n = 29) or ESC/CM (n = 31) over a total period of 28 weeks, including an acute treatment phase within the first eight weeks. Of the n = 59 patients who began treatment, n = 6 discontinued it before the end of the acute treatment phase, resulting in n = 53 completers (n = 27 CBASP; n = 26 ESC/CM), who were included in the present moderator analysis. Notably, study discontinuation occurred due to motivational or logistical reasons (e.g. move to another city, start of another therapy). We did not detect any patients in the ESC/CM group who discontinued study participation due to side effects from taking escitalopram.

The utilized statistical procedure [25] of this analysis preselects and combines multiple individual baseline variables into one optimal composite moderator (M*) to detect possible subgroup effects. Baseline variables were assessed by evaluators blinded to the treatment condition. For being preselected for the compilation of M*, a baseline variable had to contain at least n = 50 valid cases or no more than three missing cases so as not to reduce the sample size relevant for the final regression analysis. Our set of preselected initial baseline variables thus comprised 11 baseline variables from a wide range of domains (see Table 1).

**Table 1:** List of initially considered baseline variables

Baseline variable	Type	Definition/ assessment
Socio-demographic characteristics		
1. Female gender	Nominal	Yes/no
2. Age	Metric	Years
Clinical characteristics		
3. Early illness onset	Nominal	Defined as an onset of PDD before the age of 21; yes/ no
4. Depression severity	Metric	Clinician-rated MADRS total score at baseline
5. History of suicidality	Metric	Self-reported number of previous suicide attempts
6. Comorbidity of ≥ 1 Axis-I disorder	Nominal	Yes/no; diagnosed with the SCID-I by clinician
7. Comorbidity of ≥ 1 Axis-II disorder	Nominal	Yes/no; diagnosed with the SCID-II by clinician
Childhood and life trauma		
8. Childhood trauma	Nominal	Self-reported moderate-to-severe childhood trauma that occurred before the age of 18 in at least one of the five dimensions of the CTQ; yes/ no
9. Adverse life events	Metric	Item assessing the number of self-reported major psychosocial stressors over the lifetime
Previous treatments		
10. Previous psychotherapies	Ordinal	Self-reported number of previous psychotherapies, provided in categories (0 = none, 1 = 1, 2 = 2, 3 = 3, 4 = 4, 5 = more than 5)
11. Previous medication	Ordinal	Self-reported number of previous treatments with antidepressants, provided in categories (0 = none, 1 = 1, 2 = 2, 3 = 3, 4 = 4, 5 = more than 5)

*Abbreviations*: *CTQ,* Childhood Trauma Questionnaire [47]; *MADRS*, Montgomery-Asberg Depression Rating Scale [48]; *PDD,* persistent depressive disorder; *SCID-I,* Structured Clinical Interview for DSM-IV Axis I Disorders [49]; *SCID-II,* Structured Clinical Interview for DSM-IV Axis II Personality Disorders [50]

### Main outcome

The main outcome in this secondary analysis was the percentage change in MADRS scores from baseline to week eight (corresponding to the end of the acute treatment phase) calculated according to the following equation:

$$percentchang{\text{e}}_{MADRS} = \frac{{MADRS_{week 8 } - MADRS_{baseline} }}{{MADRS_{baseline} }} \times { 1}00\%$$

Based on this equation, negative values of this outcome reflect a reduction in depression severity, a score of zero reflects no change and positive scores indicate an increase in depression severity from baseline to week eight. The MADRS ratings were performed by trained and experienced evaluators. All n = 53 completers had valid MADRS scores at week eight.

### Statistical analyses

All analyses described in the following were performed in the sample of treatment completers (n = 53) at week eight using STATA version 15.1 (StataCorp 2017). To ensure that the results of the analyses were not driven by possible outliers, both the outcome variable as well as all analyzed baseline variables were tested for outliers and skewness before calculating the moderator effect sizes. We detected no outliers.

#### Calculating individual moderator effect sizes

By using the method described by Kraemer [25], we first computed moderator effect sizes for the 11 preselected baseline variables. For this, we paired each patient assigned to CBASP to each patient assigned to ESC/CM. Next, for each pair of this dataset, we calculated the difference in outcome (i.e., the percentage change in MADRS scores) and the average value of each of the 11 baseline variables. Next, for obtaining moderator effect sizes, non-parametric Spearman correlations between the difference in outcome and each average were calculated together with their 95% bootstrap confidence intervals based on 100 replications. In principle, moderator effect sizes based on this method are invariant over linear transformations of the baseline variable or the outcome, and vary between −1 and + 1, with higher magnitudes indicating a stronger moderation and zero indicating the absence of a moderation effect [25]. Baseline variables were preselected to be included in the model for complying M* when their effect size was ≥ |0.20|. This cutoff is more rigorous than others used in previously published applications of Kraemer’s composite moderator method [9, 26, 27], and was chosen as such in order to select as few meaningful moderator variables as possible to account for the modest sample size. We abstained from calculating and including statistical significance of interaction effects between the treatment variable and the baseline variables as a further selection criterion for a baseline variable to be used for the compilation of M* [28].

#### Model selection of the composite moderator

Next, we determined the statistical weights of those baseline variables with effect sizes ≥ |0.20| for inclusion in the composite moderator. For this, in the paired dataset, the weights of the single moderators were estimated by a multivariable regression model, in which the difference in outcome was predicted by the averages of all preselected variables. Analogous to previous applications of the composite moderator approach [9, 27, 29], we performed a least absolute shrinkage and selection operator (lasso) regression [30] for the multivariable model. In principal, lasso regression selects the most useful independent variables and shrinks the regression weights of the least useful variables with little predictive power or correlated with other predictors to zero, thereby removing them from the model [30]. We chose to apply this method in order to circumvent subjective and arbitrary decisions by the researchers about which variables to remove from the model.

In addition and in line with previous applications of the composite moderator method [9, 31, 32], for further optimizing the model’s predictive performance and avoiding overfitting, we combined lasso regression with *k*-fold cross-validation [33]. The methodological advantages of combining lasso regression with *k*-fold cross-validation have been explained before [e.g., 14]. Briefly, empirically based statistical methods can sometimes utilize chance associations within a single data set, making it difficult to replicate results across various studies. To protect against the exploitation of random associations, we used the *k*-fold cross-validation method. In *k*-fold cross-validation, the data is randomly sampled into *k* folds, whereby (*k*-1) folds are used as the training dataset, and the *k*th fold constitutes the validation dataset. The model is estimated within the training dataset, and its predictive performance is assessed within the held-out validation dataset [33]. The entire procedure is repeated *k* times so that each fold is used for validation once. When applied to lasso regression, *k*-fold cross-validation can be used to identify the value of the tuning parameter (λ) that minimizes the estimated mean-squared prediction error (MSPE) in the validation dataset. Thus, *k*-fold cross-validation enables the researcher to select a model that is more likely to have a good predictive performance in future new data, than a model that was trained and tested within the same data. Given the modest sample size and the associated need to replicate the results provided in our study, we decided to use this method in order to provide more reproducible results.

Concretely, in our analysis, for defining the optimal tuning parameter that yields the smallest MSPE, we applied 10-folds cross-validation as described by Ahrens et al. [34] and implemented in their package *“lassopack”* developed for use in STATA. Within the paired dataset, we ran the 10-folds cross-validation by using the command “*cvlasso*”, which internally repeats lasso regression and finally selects the model with the optimal tuning parameter (λ_opt_) that yields the smallest MSPE.

#### Identification and characterization of subgroups

After selecting the optimal model based on the procedure described before, weights from each of the moderators selected by this model were extracted in order to calculate the value of M* for each patient as described by Kraemer [34]. Thereafter, in the unpaired dataset, we performed a regression analysis predicting the outcome (i.e., percentage change in MADRS scores) from the composite moderator M*, the treatment group, and their interaction. We furthermore computed the moderator effect size of the composite moderator M* together with its 95% bootstrap confidence interval. We then calculated the value of M* at which the predicted outcomes for the CBASP and ESC/CM group intersected and divided the sample into two subgroups, one below and one above this cross-point. Each of these subgroups is consequently associated with a likely more beneficial outcome for one of the two treatments compared to the other. For characterizing and comparing the two identified subgroups, we analyzed and compared relevant baseline characteristics and calculated between-group treatment effect sizes (Cohen’s *d*).

#### Subgroup and treatment interaction effects

We next analyzed whether those patients who received their likely more beneficial treatment condition had higher response and remission rates than those who received their likely less beneficial condition. For this, we stratified the sample of completers in four clusters: 1. Patients randomized to CBASP and likely to respond better to CBASP; 2. Patients randomized to CBASP and likely to respond better to ESC/CM; 3. Patients randomized to ESC/CM and likely to respond better to ESC/CM; 4. Patients randomized to ESC/CM and likely to respond better to CBASP. In these four clusters, we compared rates of response (defined as ≥ 50.0% reduction in MADRS scores from baseline to week eight) and remission (defined as a MADRS score of  ≤ 9 at week eight). We also analyzed between-cluster differences in MADRS scores at week eight as well as values of the percentage change of the MADRS scores from baseline to week eight. Finally, we examined whether those patients who did not experience a change of at least 20.0% after the acute treatment phase and who received augmentation with the other treatment condition were, in majority, those who did not receive their likely more beneficial treatment condition during the acute treatment phase.

## Results

### Effect sizes of individual moderators

Among the 11 tested baseline variables, we identified six with an effect size < 0 and five with an effect size > 0. Negative values indicate a better outcome (i.e., a greater percentage reduction in MADRS scores from baseline to week eight) with ESC/CM than with CBASP for higher values or the presence of that moderator. Positive values indicate a better outcome with CBASP than with ESC/CM for higher values or the presence of that moderator. Individual moderator effect sizes, 95% confidence intervals and lasso regression coefficients derived from the lasso regression model are displayed in Table 2.

**Table 2:** Moderator effect sizes, 95% confidence intervals and lasso regression coefficients for selected and deselected baseline variables

Baseline variables	Effect size	95% CI	Lasso coefficient^a^
**Indicating a superiority of ESC/CM**			
* Selected*			
Number of previous suicide attempts	−0.363	(−0.429; −0.297)	–8.806
Adverse life events	−0.288	(-0.354; -0.222)	−9.782
Childhood trauma^b^	−0.251	(−0.326; −0.175)	−65.803
Female gender	−0.213	(−0.280; −0.146)	−31.344
*Not selected*			
MADRS (baseline) score	−0.117	(−0.188; −0.047)	
Comorbidity with ≥ 1 Axis-II disorder	−0.063	(−0.144; 0.018)	
**Indicating a superiority of CBASP**			
*Selected*			
Age	0.238	(0.166; 0.309)	2.817
Early illness onset	0.215	(0.146; 0.285)	56.434
Number of previous treatments with AD	0.212	(0.155; 0.269)	12.629
* Not selected*			
Number of previous psychotherapies	0.085	(0.025; 0.145)	
Comorbidity with ≥ 1 Axis-I disorder	0.042	(−0.016; 0.100)	

^a^Displayed only for those variables selected by the final lasso regression model; coefficients indicate the weight in the composition of M* as derived from the lasso regression model

^b^Presence indicates a clinical severity of at least moderate-to-severe on at least one of the five dimensions of the CTQ

*Abbreviations*: *AD,* antidepressants; *CI,* confidence interval; *CTQ,* Childhood Trauma Questionnaire [47]; *MADRS,* Montgomery-Asberg Depression Rating Scale [48]

The strongest moderator indicating a superiority of ESC/CM was a higher number of previous suicide attempts (effect size = −0.36); the strongest moderator indicating a superiority of CBASP was a higher age (effect size = 0.24). In total, we identified seven baseline variables with an effect size ≥ |0.20|. These were: number of previous suicide attempts, number of adverse life events, the presence of at least one form of moderate-to-severe childhood trauma, age, an early illness onset, female gender, and the number of previous treatments with antidepressants (for effect sizes, see Table 2). These seven variables were further used to calculate the composite moderator M*.

Other analyzed baseline variables whose moderator effect sizes were below the selected threshold (r < |0.20|) and which were therefore not included in the calculation of the composite moderator were baseline depression severity, comorbidity with at least one Axis-I or Axis-II disorder, and the number of previous psychotherapies (see Table 2).

### Composite moderator

The lasso regression yielded lasso coefficients for all seven selected baseline variables (see Table 2, right column), which were all further combined to develop the composite moderator M*. The lasso coefficients represent the extent to which each baseline variable distinguishes differences in the outcome between patients treated with ESC/CM and those treated with CBASP in the context of the other selected variables. The composite moderator M* was calculable for n = 50 patients who had complete data on all seven variables. Table 3 provides descriptive statistics for this sample of patients with values for M*. With r = 0.67 (95% CI: 0.63; 0.71), the effect size of M* was larger than any effect size of the individual baseline variables.

**Table 3:** Descriptive statistics for the identified subgroups

Variables	Entire sample of patients with values for M* (n = 50)	Subgroup likely to benefit more from ESC/CM than from CBASP (n = 28)	Subgroup likely to benefit more from CBASP than from ESC/CM (n = 22)	Difference between subgroups with 95% CI and *p*-value
Baseline variables which were included in the compilation of M*				
Female gender (%)	50.0	60.7	36.4	24.4 (−2.7; 51.4); *p* = 0.2
Age in years (mean (SD))	42.9 (1.08)	40.2 (10.8)	46.4 (9.9)	−6.2 (−12.2; −0.2); *p* = 0.04
Early illness onset (%)	58.0	42.9	77.3	−34.4 (−59.8; −9.1); *p* = 0.02
Number of previous suicide attempts (mean (SD))	0.3 (0.7)	0.50 (0.8)	0.1 (0.4)	0.4 (0.03; 0.8); *p* = 0.03
Childhood trauma (%)^a^	70.0	82.1	54.5	27.6 (2.4; 52.8); *p* = 0.06
Number of adverse life events (mean (SD))	1.8 (1.1)	2.1 (1.2)	1.4 (0.8)	0.7 (0.1; 1.4); *p* = 0.02
Proportion of patients with following frequency of previous treatments with AD (%)				
n = 0	44.0	46.4	40.9	Difference in chances of having ≥ 1 previous treatment with AD: −5.5 (−33.1; 22.1); *p* = 0.8
n = 1	20.0	25.0	13.6	
n = 2	8.0	7.1	9.1	
n = 3	18.0	17.9	18.2	
n = 4	4.0	3.6	4.6	
n ≥ 5	6.0	0.0	13.6	
Further variables which were not included in the compilation of M*				
MADRS baseline score (mean (SD))	26.6 (8.6)	28.4 (8.1)	24.4 (8.8)	4.1 (−0.8; 8.9); *p* = 0.10
Diagnosis of ≥ 1 comorbid Axis-I disorder (%)^b^	48.0	57.1	36.4	20.8 (−6.4; 48.0); *p* = 0.2
Diagnosis of ≥ 1 comorbid Axis-II disorder (%)^c^	38.0	39.3	36.4	2.9 (−24.1; 30.0); *p* = 1.00
History of ≥ 1 suicide attempt (%)	22.0	35.7	4.5	31.2 (11.4; 50.9); *p* = 0.01
Emotional abuse (%)^d^	38.0	39.3	36.4	2.9 (−24.1; 30.0); *p* = 1.00
Physical abuse (%)^d^	12.0	14.3	9.1	5.2 (−12.5; 22.9); *p* = 0.7
Sexual abuse (%)^d^	10.0	14.3	4.5	9.7 (−5.9; 25.3); *p* = 0.4
Emotional neglect (%)^d^	56.0	57.1	54.5	2.6 (−25.1; 30.0); *p* = 1.00
Physical neglect (%)^d^	34.0	35.7	31.8	3.9 (−22.4; 30.2); *p* = 1.00
Proportion of patients with following frequency of previous psychotherapeutic treatments (%)				
n = 0	30.0	32.1	27.3	Difference in chances of having ≥ 1 previous psychotherapy: −4.9 (−30.3; 20.5); *p* = 0.8
n = 1	24.0	28.6	18.2	
n = 2	20.0	17.9	22.7	
n = 3	10.0	10.7	9.1	
n = 4	6.0	7.1	4.6	
n ≥ 5	10.0	3.6	18.2	
Response at week eight (%)	20.0	25.0	13.6	11.4 (−10.2; 32.9); *p* = 0.5
Remission at week eight (%)	12.0	14.3	9.1	5.2 (−12.5; 22.9); *p* = 0.7

*Note:* For nominal and ordinal variables, *p*-values and 95% CIs from the Fisher’s exact tests are reported. For metric variables, *p*-values and 95% CIs from independent sample *t*-tests are reported

^a^Presence indicates a clinical severity of at least moderate-to-severe on at least one dimension of the CTQ

^b^Prevalences (%) for comorbid Axis-I disorders were as follows: Alcohol abuse: 10.0; Substance abuse: 4.0; Panic disorder: 4.0; Panic disorder with agoraphobia: 8.0; Social phobia: 30.0 (46.7 CBASP benefit); Specific phobia: 10.0; Generalized anxiety disorder: 2.0; Bulimia nervosa: 2.0; Binge eating disorder: 4.0; all other Axis-I disorders: 0.0

^c^Prevalences (%) for comorbid Axis-II disorders were as follows: Self-insecure personality disorder: 26.0 (38.5 CBASP benefit); Obsessive–compulsive personality disorder: 12.0; Depressive personality disorder: 4.0; Paranoid personality disorder: 4.0; all other Axis-II disorders: 0.0

^d^Presence indicates a clinical severity of at least moderate-to-severe on the respective dimension of the CTQ

*Abbreviations*: *AD,* antidepressants; *CBASP,* Cognitive Behavioral Analysis System of Psychotherapy; *CI,* confidence interval; *CTQ,* Childhood Trauma Questionnaire [47]; *ESC/CM,* escitalopram plus clinical management; *MADRS,* Montgomery-Asberg Depression Rating Scale [48]; *SD,* standard deviation

**Fig. 1:**
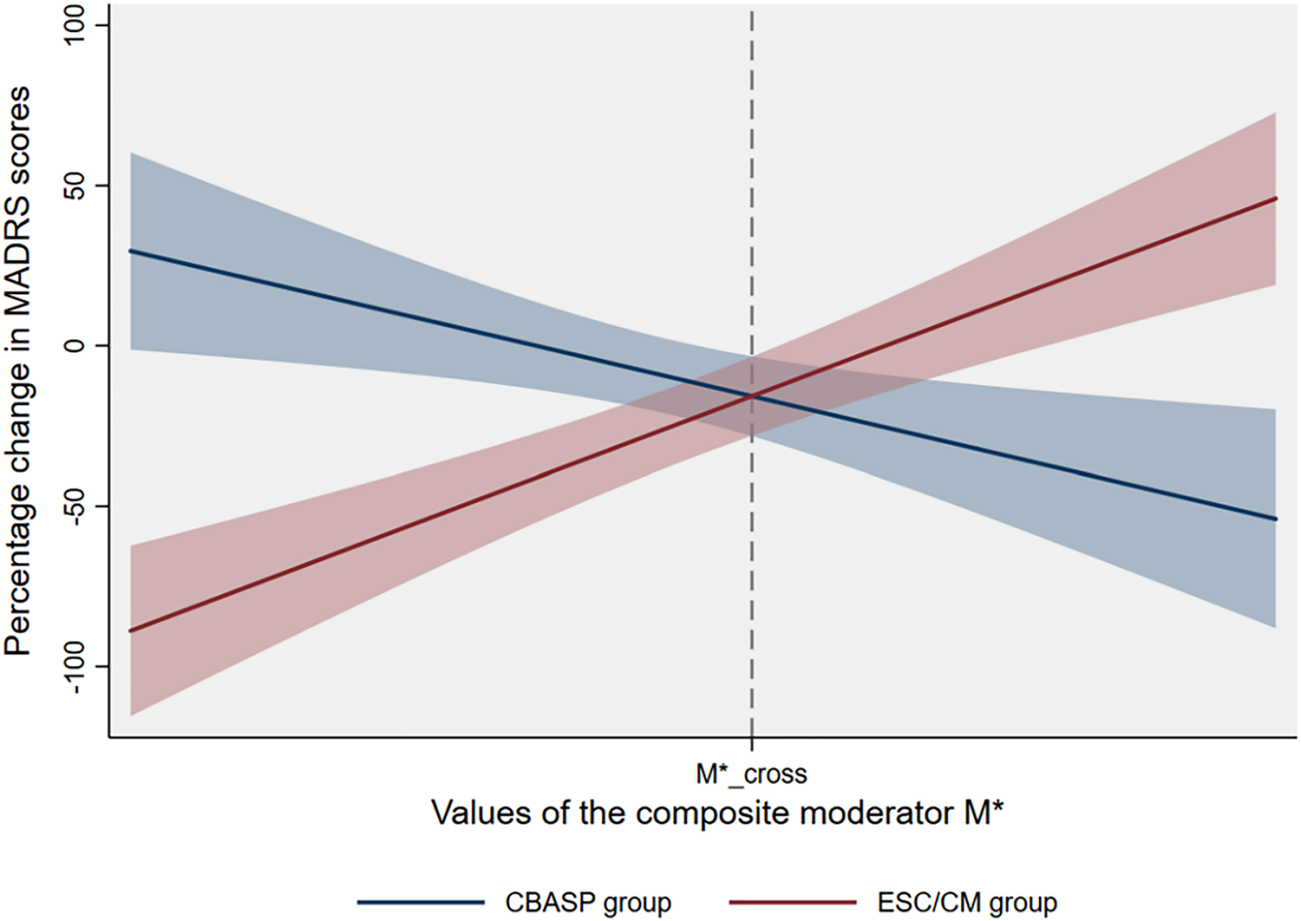
Predicted percentage reduction in MADRS scores with 95% confidence intervals for CBASP and ESC/CM across the range of the composite moderator M*. Negative values of the y-axis reflect a desired reduction in depression severity from baseline to week eight, a score of zero reflects no change, and positive scores indicate an increase in depression severity from baseline to week eight. *Abbreviations:* *CBASP,* Cognitive Behavioral Analysis System of Psychotherapy, *ESC/CM* escitalopram plus clinical management, M* composite moderator, *MADRS*, Montgomery-Asberg Depression Rating Scale

In the unpaired dataset, we next performed a simple regression analysis as explained in the methods. The final regression model revealed a statistically significant interaction effect between the treatment variable and M* in predicting the individual-participant MADRS percentage change values (interaction term β = 0.95, S.E. = 0.17, *p* < 0.001, *R*^2^ = 0.48). Figure 1 illustrates the predicted percentage change in MADRS scores from baseline to week eight for the CBASP and ESC/CM treatment groups across the observed range of M* with 95% confidence intervals. For n = 28 (56.0%) of the n = 50 patients who scored below this cross-point (M* < 46.94), treatment with ESC/CM was associated with a likely better outcome (i.e., greater percentage reduction in MADRS scores) compared to treatment with CBASP (Cohen’s *d* = −1.76; 95% CI: −2.64; −0.86). For n = 22 (44.0%) of the n = 50 patients who scored above this cross-point (M* > 46.94), treatment with CBASP was associated with a likely better outcome compared to treatment with ESC/CM (Cohen’s *d* = 1.28; 95% CI: 0.33; 2.19).

### Baseline profiles of identified subgroups

We next compared the baseline profiles of the two identified subgroups. Table 3 presents descriptive statistics for all seven baseline variables used to create the composite moderator M* per subgroup. To provide an even more comprehensive picture of the profiles of the two subgroups, Table 3 also shows descriptive statistics for additional baseline variables that were not included in the calculation of M*, as well as for response and remission rates at week eight. To provide guidance on which differences between the subgroups are significant, differences in means or percentages are reported along with the corresponding 95% confidence intervals and *p*-values determined by statistical significance testing. Importantly, due to the modest sample size and exploratory nature of this analysis, reported differences between the subgroups will be discussed with caution.

#### Description of the subgroup likely to benefit more from ESC/CM

In comparison with the subgroup likely to benefit more from CBASP, patients in the subgroup likely to benefit more from ESC/CM were more often female (60.7% versus 36.4%) and had a higher average number of previous suicide attempts (average number of 0.5 versus 0.1). This goes in line with this subgroup reporting more often at least one previous suicide attempt (35.7% versus 4.5%). Moreover, patients in this subgroup reported more often at least one form of moderate-to-severe childhood trauma (82.1% versus 54.5%) as well as more adverse life events (average number of 2.1 versus 1.4).

#### Description of the subgroup likely to benefit more from CBASP

In comparison to the subgroup of patients likely to benefit more from ESC/CM, those likely to benefit more from CBASP tended to be slightly older (mean age of 46.4 years versus 40.2 years) and had more often an early illness onset (i.e., before the age of 21; 77.3% versus 42.9%). The previous usage of antidepressant medication was slightly higher in this subgroup: 59.1% (versus 53.6% in the other subgroup) of the patients in this subgroup had taken antidepressant medication at least once and 13.6% (versus 0.0% in the other subgroup) reported more than five previous treatments with antidepressants.

#### Further analyzed baseline variables

As displayed in Table 3, we did not detect statistically significant differences between the two subgroups with respect to the rates of female gender, childhood trauma and previous treatments with antidepressant medication (all *p* > 0.05). Further, except for the history of at least one suicide attempt, which was not selected for the compilation of M* because of the intercorrelation with the mean number of suicide attempts, none of the baseline variables deselected for the compilation of M* showed significant differences between the subgroups. Consequently, the two subgroups were relatively similar with respect to the MADRS mean baseline scores, rates of comorbid Axis-I and Axis-II diagnoses, various subtypes of childhood trauma assessed by the CTQ (i.e., emotional abuse, sexual abuse, physical abuse, emotional neglect, and physical neglect), and previous numbers of underwent psychotherapies. The same was true for both response and remission rates at week eight.

### Differences in outcomes for each subgroup by treatment

**Fig. 2:**
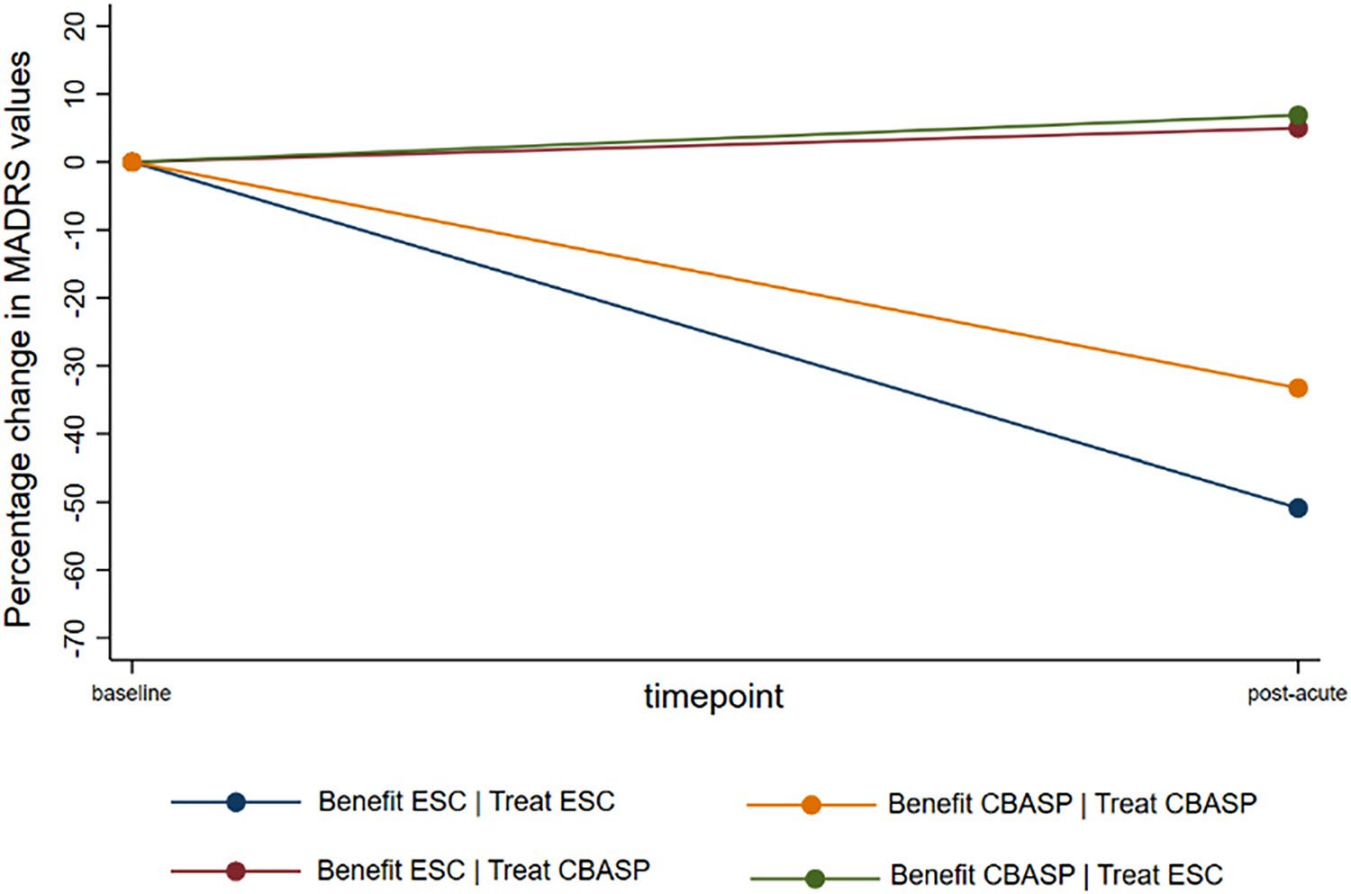
Percentage change in MADRS values for each subgroup by randomized treatment. *Abbreviations:* *CBASP,* Cognitive Behavioral Analysis System of Psychotherapy; *ESC/CM,* escitalopram plus clinical management; *MADRS,* Montgomery-Asberg Depression Rating Scale [48]

In the subgroup likely to benefit more from ESC/CM, n = 12 patients underwent treatment with ESC/CM, while n = 16 received treatment with CBASP, which was likely less effective for them. In the subgroup likely to benefit more from CBASP, n = 10 patients underwent treatment with CBASP, while n = 12 received treatment with ESC/CM, which was likely less effective for them. Table 4 shows MADRS mean values at baseline and at week eight, as well as the mean percentage change and rates of response and remission at week eight for each of the four subgroups by randomized treatment condition. It also shows the same outcomes for those patients who received their likely more beneficial treatment and for those who received their likely less beneficial treatment. Briefly, we can conclude that patients likely to benefit more from ESC/CM and treated with ESC/CM had the largest percentage decrease (-50.9%) in depression severity from baseline to week eight, as well as the highest response (58.3%) and remission rates (33.3%) at week eight. They are followed by patients likely to benefit more from CBASP and treated with CBASP, which show more modest values in terms of percentage decrease (-33.3%) in depression severity as well as response (20.0%) and remission (10.0%) rates. Patients likely to benefit more from CBASP and treated with ESC/CM had, in average, an increase in depression severity (6.9%) from baseline to week eight and relatively low response and remission rates (both 8.3%) at week eight. Depression severity increased on average (5.0%) also among patients likely to benefit more from ESC/CM and treated with CBASP; additionally, there were no remitters or responders in this subgroup. The average percent change in depression severity from baseline to week eight is illustrated in Fig. 2 for each subgroup by treatment interaction.

**Table 4:** Comparison of different outcomes for each subgroup by assigned treatment

Subgroup x treatment	MADRS at baseline, mean (SD)	MADRS at week 8, mean (SD)	Mean change (%, SD)	Response at week 8, %	Remission at week 8, %
Be ESC/CM|Tr ESC/CM; n = 12	30.1 (6.9)	15.1 (11.2)	−50.9 (35.4)	58.3	33.3
Be CBASP|Tr CBASP; n = 10	26.8 (8.5)	17.5 (6.9)	−33.3 (22.3)	20.0	10.0
Be CBASP|Tr ESC/CM; n = 12	22.3 (8.9)	22.4 (9.4)	6.9 (37.3)	8.3	8.3
Be ESC/CM|Tr CBASP; n = 16	27.2 (9.0)	27.3 (8.4)	5.0 (28.7)	0.0	0.0
Be ESC/CM|Tr ESC/CM + Be CBASP|Tr CBASP; n = 22	28.6 (7.6)	16.2 (9.4)	−42.9 (30.8)	40.9	22.7
Be ESC/CM|Tr CBASP + Be CBASP|Tr ESC/CM; n = 28	25.1 (9.1)	25.2 (9.0)	5.8 (32.0)	3.6	3.6

*Abbreviations*: *Be* [treatment condition] = likely higher benefit from this treatment condition; *CBASP,* Cognitive Behavioral Analysis System of Psychotherapy; *ESC/CM,* escitalopram plus clinical management; *MADRS,* Montgomery-Asberg Depression Rating Scale [48]; *SD,* standard deviation, *Tr* [treatment condition]= treated with this treatment condition

When pooling those patients who received their likely more beneficial treatment, the average decrease in depression severity reached up 42.9%, while response and remission rates were 40.9% and 22.7%, respectively. These numbers stand in contrast to the pool of patients who received their likely less beneficial treatment, having an average increase in depression severity of 5.8% and smaller remission and response rates of 3.6%.

### Sub-analysis of patients augmented after week eight

As mentioned above, after completion of the 8-week acute treatment phase, a total of n = 20 patients whose depression severity had not decreased by at least 20.0% received the other treatment condition in addition to the initial treatment condition for the following 20 weeks. The main analysis showed that these patients eventually caught up with the initial improvers in terms of depression scores by the end of the extended treatment phase, reaching a remission rate of 30.0% and a response rate of 45.0% [22]. However, considering the present analyses enabling the stratification in subgroups based on the composite moderator, we can conclude that n = 19 (95.0%) of these n = 20 patients initially received their less beneficial treatment: 50.0% would likely have benefited more from ESC/CM and received CBASP and 45.0% vice versa. Only one patient who likely benefited more from ESC/CM and who received this treatment experienced no reduction of at least 20.0% and was supplemented with CBASP after week eight.

## Discussion

### Identified subgroups

The aim of our study was to identify and characterize subgroups of patients who were likely to benefit more from psychotherapy with CBASP than from ESC/CM or vice versa during an eight-week acute treatment phase. In summary, we can conclude that below the general lack of statistically significant differences in the effectiveness of these two treatments observed in the main analysis [22], there are however considerable subgroup effects implying that in terms of reduction in depression severity, certain patients did not benefit from their assigned condition within the first eight weeks.

Notably, with r = 0.67 (95% CI: 0.63; 0.71), the effect size of M* was larger than any effect size reported by comparable studies (e. g., r = 0.41 [27]; r = 0.34 [9]; r = 0.31 [16]; r = 0.29 [32]; r = 0.28 [32]; r = 0.20 [31]), thereby reflecting a large moderation effect and difference in beneficial responses of the subgroups to the two investigated treatments.

### Patients augmented after week eight

In addition, we found that patients who did not achieve at least a 20.0% reduction in symptom severity after the acute treatment phase and who were subsequently supplemented with the other treatment condition were in the majority (95.0%) treated with their likely less effective treatment during the acute treatment phase. The main analyses [22] found that these patients benefited substantially from the addition of the other treatment condition, resulting in response rates of 45.0% and remission rates of 30.0% at the end of the extended treatment phase. This subsequent improvement during the extended treatment phase can be plausibly explained by the fact that after week eight, these patients received the treatment that was likely more effective for them personally in addition to the first, unsuccessful treatment, rather than that they received an additional treatment per se. This reasoning may also contribute to explain why there is no clear empirical evidence as to whether the combination of medication and psychotherapy always works better than monotherapy in patients with PDD, as shown by a review of Spijker et al. [35].

### Baseline differences between the identified subgroups

#### Patients benefiting more from ESC/CM

Although the modest sample size of our study does not allow us to draw general conclusions about the pre-treatment profile of the identified subgroups, we can nevertheless summarize some interesting trends that have been uncovered: to sum up, the subgroup likely to benefit more from ESC/CM was more often female, reported more often at least one form of moderate-to-severe childhood trauma as well as more previous suicide attempts and more adverse life events. Similarly, a traditional moderator analysis of this trial by Bausch et al. [17] found that patients with moderate-to-severe childhood trauma responded and remitted more often to ESC/CM than to CBASP within the first eight weeks of treatment. Further, patients reporting moderate-to-severe childhood trauma tended to have more previous suicide attempts in our sample [17], which is in line with other findings: for instance, childhood trauma particularly in the form of physical abuse or sexual abuse has been reported to enhance the risk for suicidal attempts later in life in the general population [36], while childhood emotional trauma was reported as a predictor for an elevated suicide risk in patients with major depression [37]. Besides higher levels of childhood trauma, female gender was also found to be an independent risk factor for both an early onset of first attempting suicide and for a higher number of suicidal attempts [38]. A study by Sarchiapone et al. [39] conducted in patients with unipolar depression revealed that being female, having childhood trauma as well as a lifetime history of aggression significantly increased the risk of previous suicide attempts. Taken together, these previous findings suggest an association between female gender, childhood trauma and possibly also later traumatic events, as well as suicide attempts, which is complemented by the results of our analyses in that this phenotype may benefit better from medication with escitalopram in the acute treatment phase than from CBASP in the context of PDD. Notably, this interpretation is further supported by the fact that in our sample, patients reporting moderate-to-severe childhood trauma reported significantly more often resistances to treatments with psychotherapy, indicating that for some patients, psychotherapy has also failed to lead to a response in the past.

Moreover, a possible explanation for the poorer response of early traumatized patients to CBASP in our trial could be that the invocation of memories of early traumatic experiences through CBASP may have led to an initial worsening of symptoms in these patients within the first eight weeks of treatment [17]. When treated with CBASP, PDD patients with moderate-to-severe childhood trauma may need a longer treatment time in order to cognitively restructure traumatic memories as well as to establish healthier interpersonal behavioural patterns and thereby recover from PDD [4, 40, 41]. Combining CBASP from the beginning of treatment with escitalopram or a comparable antidepressant could help early traumatised patients to cope with the mental and emotional consequences of recalling and processing past traumatic experiences. The improvement observed in some of these patients after augmentation with ESC/CM in the extended treatment phase supports this assumption.

Furthermore, our findings complement a recent systematic review and meta-analysis by Kuzminskaite et al. [42], who found that, in contrast to previous studies, patients with major depressive disorder and childhood trauma benefited from active treatments similarly to patients without childhood trauma, despite their higher severity of depressive symptoms at baseline. While this meta-analysis suggests that evidence-based psychotherapy and pharmacotherapy should be offered to patients with major depressive disorder regardless of their childhood trauma status, our findings rather point in the direction that the differential benefits that patients with childhood trauma may derive from pharmacotherapy versus psychotherapy and its combination should be further investigated.

#### Patients benefiting more from CBASP

In contrast, patients likely to benefit more from CBASP than from ESC/CM had more often an early illness onset. Given that CBASP was especially developed to meet the needs of patients with PDD with an early illness onset [43], it is plausible that its specific techniques to address early onset based symptoms and illness-trajectories have led to a greater reduction in depression severity in these patients. The patients in this subgroup were also older, which is in line with a meta-regression by Furukawa et al. [8], which revealed that younger PDD patients discontinued monotherapy with CBASP more often across three large studies including this RCT, possibly because of a lack of response or acceptance of CBASP.

Furthermore, patients in this subgroup reported more previous treatments with antidepressant medication, and also more often treatment resistances to previous treatments with antidepressants (53.8% in this subgroup versus 13.3% in the subgroup benefiting more from ESC/CM). The higher rate of previous treatment resistances to antidepressants and the fact that these patients participated in our trial suggests that previous medication therapies did not lead to responses, long-term remission or prevention of relapses, which may be due to a reduced neurobiological and/or metabolic responsiveness to antidepressants in these patients [44–46] and could explain their poorer response to ESC/CM in our trial. Note that in contrast to the rates of reported resistance to past antidepressant medication, those to previous treatments with psychotherapy were similar in both subgroups (13.3% in the subgroup benefiting more from CBASP versus 15.8% in the subgroup benefiting more from ESC/CM).

#### Further analysed baseline variables

We did not detect statistically significant differences between the two subgroups with respect to rates of female gender, moderate-to-severe childhood trauma and previous treatments with antidepressant medication (all *p* > 0.05, see Table 3), although these baseline variables showed large moderator effect sizes (see Table 2). However, the lack of statistical significance can presumably be explained by the small sample size and highlights the importance of effect sizes for this type of exploratory analyses, particularly when being performed based on modest sample sizes. Finally, the two subgroups were relatively similar in terms of the baseline depression severity (MADRS mean score), all subtypes of childhood trauma as well as the previous numbers of underwent psychotherapies and rates of comorbid Axis-I and Axis-II diagnoses. Notably, most of the distinct Axis-I and Axis-II disorders were present in very small numbers of cases, with social phobia being the most common Axis-I disorder with a prevalence of 30.0% and self-insecure personality disorder the most common Axis-II disorder with 26.0% (see Table 3). No significant differences were found in the distribution of these distinct comorbid disorders between the two subgroups. In sum, these baseline variables may or may not play a role as predictors of treatment efficacy, which warrants investigation in further analyses.

### Strengths and limitations

The results of our study should be viewed considering certain strengths and limitations:

#### Study strengths

In terms of strengths, first, we compared two clinically highly relevant treatments in terms of their efficacy for specific subgroups of outpatients with PDD. Second, based on the composite moderator method, we generated findings about the influence of numerous, for replication studies relatively easy to assess baseline variables, instead of examining the effect of only one potential moderator. Third, the design of the original study furthermore allowed us to examine the relationship between subgroup classification, initial treatment, and the effect of a combination treatment after the acute 8-week treatment phase.

#### Study limitations

Nevertheless, some important limitations should be considered as well: first, due to the initial sample size and the lack of baseline data in some patients, we had a relatively modest sample size at the basis of our analyses. This factor results in relatively small cell numbers when comparing both subgroups, which is why the comparison of the baseline profiles should be interpreted with caution. In this light, it is important to emphasize that the identified trends must be confirmed in further independent studies with larger populations and more participating centers before any treatment recommendations can be drawn. Second, the composite moderator was based on the set of available baseline variables and is thus one of many possible. Very likely, there were other not assessed relevant moderators such as genetic or neural biomarkers, that could have helped to further differentiate the subgroups. Moreover, other outcomes relevant to treatment success in PDD such as improvements of life quality or interpersonal relationships could be analysed in further secondary analyses. Finally, our study had specific inclusion and exclusion criteria, so the generalizability of these results remains to be verified.

## Conclusion

This study has highlighted the impact of several important features of the baseline profile of patients with PDD on their response to an acute psychotherapeutic versus pharmacological treatment. After being confirmed in independent studies, these findings could serve to inform clinical decision-making by helping clinicians to assign the most promising treatment to individual patients based on their baseline profile. The progress of personalized medicine could, together with the development of new therapies, substantially improve the life of individuals affected by PDD.

## Data Availability

The data that support the findings of this study are not openly available due to reasons of sensitivity and anonymity of the study participants.
